# Association between physical activity and dynapenia in older adults with COPD: a nationwide survey

**DOI:** 10.1038/s41598-022-11504-1

**Published:** 2022-05-06

**Authors:** Young-Ah Choi, Jung Soo Lee, Yeo Hyung Kim

**Affiliations:** 1 Department of Rehabilitation Medicine, Incheon St. Mary’s Hospital, College of Medicine, The Catholic University of Korea, Seoul, Republic of Korea; 2 Department of Rehabilitation Medicine, College of Medicine, The Catholic University of Korea, Seoul, Republic of Korea

**Keywords:** Chronic obstructive pulmonary disease, Risk factors

## Abstract

We aimed to examine the association between physical activity (PA) level and dynapenia in older adults with chronic obstructive pulmonary disease (COPD), and whether it varied with sex and obesity status. The current cross-sectional study included total of 1033 community-dwelling participants with COPD aged 65–79 from the Korean National Health and Nutrition Examination Survey. In the multivariable model, high and moderate PA levels were significantly associated with lower odds of dynapenia than low PA levels (high PA level: odds ratio [OR] = 0.26, 95% confidence interval [CI] = 0.09–0.74; moderate PA level: OR = 0.55, 95% CI = 0.35–0.87). This inverse association was observed only in males with COPD (high PA level: OR = 0.17, CI = 0.04–0.65; moderate PA level: OR = 0.49, 95% CI = 0.27–0.88) and the normal-weight group (18.5 ≤ body mass index (BMI) < 25 kg/m^2^; high PA level: OR = 0.21, 95% CI = 0.05–0.88; moderate PA level: OR = 0.48, 95% CI = 0.27–0.86). In older community-dwelling patients with COPD, a negative dose-dependent relationship exists between PA level and dynapenia. The independent associations between PA level and dynapenia was significant in men and in participants with normal weight.

## Introduction

Chronic obstructive pulmonary disease (COPD) is a prevalent pulmonary disease worldwide that causes irreversible progressive air-flow obstruction^1^. Aging is a risk factor for COPD, and the prevalence of COPD is reported to be approximately 14% among those aged over 65^2^. The multisystemic effect of COPD leads to skeletal muscle dysfunction, reduced functional exercise capacity, and decreased physical activity (PA)^3^. Therefore, COPD is a significant public health burden in contemporary years, especially in older people.

Dynapenia is defined as the loss of muscle strength associated with aging not caused by neurological or muscular disorders. Dynapenia is commonly measured using dynamometry equipment^4^. Owing to the feasibility and convenience of the handgrip strength test, dynapenia is currently used to screen and diagnose sarcopenia^5^. Dynapenia reflects age-related loss of muscle strength and is a better predictor of low physical performance or mortality than low muscle mass^6,7^. The impacts of PA on the skeletal muscle metabolism with aging have been of interest to the geriatrists^8^. Earlier researchers have emphasized numerous beneficial effects of PA including reduced morbidity, mortality, and improved quality of life^9,10^. Therefore, the World Health Organization (WHO) strongly recommends a sufficient level of aerobic PA in the older population^11^. In patients with COPD, the physical inactivity associated with the respiratory and appendicular muscles dysfunction is a significant prognostic factor of the all-cause mortality^12^.

Although both decreased PA and defective muscle function are characteristic features in patients with COPD, there have been few inconclusive previous reports on the relationship between PA level and muscular strength in older patients with COPD^13,14^. Thus, we hypothesized that there may be a negative association between PA level and dynapenia in older patients with COPD, affected by potential confounders^15–17^. Finally, the objective of the present study was to analyze the independent association between PA level and the presence of dynapenia, after adjusting for multiple potential confounders among older patients with COPD based on a nationwide representative cohort. In addition, data from earlier studies have found several important clinical confounders^18–21^. The protective effect of PA on dynapenia differs by sex^18^ and obesity is associated with muscle strength and PA^19,20^. Therefore, we performed additional stratified analyses of the association between PA level and dynapenia according to sex and obesity status to detect specific differences between subgroups.

## Methods

### Study population

This study collected data from the sixth and seventh Korea National Health and Nutrition Examination Surveys (KNHANES), which recruited participants from 2014 to 2018. The Korean Centers for Disease Control and Prevention (KCDC) operates the KNHANES and collects health and nutritional data of the community-dwelling population through house-hold interviews and examinations at mobile centers. The KNAHNES incorporates a sampling method with stratified, multi-stage, clustered probability design to gather nationally representative health information, and the data resource profiles have been previously published^22^.

The current cross-sectional study included older participants with COPD aged 65–79 who had completed spirometry, handgrip strength test, and surveys for PA and nutritional intake.

### Ethics approval

The protocol was approved by the Institutional Review Board (IRB) at KCDC. The IRB of our university exempted the study from ethical approval regulations as we used publicly accessible data. All methods were performed in accordance with the relevant guidelines and regulations.

### Measurements

Spirometry was performed in all participants by specialized technicians according to the manual of the American Thoracic Society/European Respiratory Society Task Force. COPD was defined as a forced expiratory volume in 1 s (FEV1)/forced vital capacity (FVC) < 0.7 according to the Global Initiative for Chronic Obstructive Lung Disease (GOLD) guidelines^23^. The impairment of lung function was evaluated according to the GOLD stages using FEV1% of predicted: stage 1, ≥ 80%; stage 2, 50–79%; stage 3, 30–49%; and stage 4, < 30%.

The handgrip strength was measured by the nurses using a digital dynamometer (T.K.K.5401; Takei, Niigata, Japan). It was measured thrice each for both hands in a standing posture with a neutral position of the arm, wrist, and hand^24^. The average handgrip strength of the dominant hand was analyzed as the handgrip strength of the participant^5,25^. Dynapenia was defined as the handgrip strength < 26 kg for men and < 18 kg for women using the Asian Working Group for Sarcopenia criteria^5^.

PA was assessed using the Korean version of the WHO Global Physical Activity Questionnaire (GPAQ)^26^. The GPAQ measures aerobic activities requiring physical effort that increases respiration or heart rate. The frequency (days/week) and duration (min/day) spent in a typical week performing moderate- or vigorous-intensity activities in work, travel, and leisure domains were recorded. The level of total PA was classified into high, moderate, and low levels according to the WHO guidelines^27^.

Smoking was categorized as never, past, or current smoker^28^. Excessive intake of alcohol was defined as drinking alcohol > 10 g/day for women and > 20 g/day for men^28^. Trained dieticians evaluated the nutritional intake using the 24-h recall method, and daily total caloric intake (kcal/day) was calculated. The individuals were considered as hypertensive with one of the following criteria: (1) systolic blood pressure ≥ 140 mmHg, (2) diastolic blood pressure ≥ 90 mmHg, and (3) use of blood pressure medicines. The participants were judged to have diabetes when the fasting plasma glucose level was ≥ 126 mg/dL, glycosylated hemoglobin assay with a threshold of ≥ 6.5%, diagnosed as diabetes, or when being administered a hypoglycemic agent or insulin therapy. Anemia was defined as a hemoglobin level < 13 g/dL for men and < 12 g/dL for women. Self-reported comorbidities of asthma, cardiovascular diseases, and depression were recorded.

### Statistical analyses

The complex-sample analysis of variance for continuous variables and complex-sample chi-square test for categorical variables were employed to compare participant characteristics using each PA level. The association between PA level and dynapenia was evaluated using complex-sample multivariable logistic regression analyses. Three progressive models were built during the analyses for assessing the potential confounding associations. To control the confounding effect of sex and obesity on the associations between PA level and dynapenia, stratified analyses according to sex (men and women) and obesity status (normal weight participants, 18.5 ≤ body mass index (BMI) < 25 kg/m^2^; and obese participants, BMI ≥ 25 kg/m^2^) were additionally performed. Complex sample analyses were conducted using SPSS (version 24; IBM/SPSS Inc., Armonk, NY, USA).

## Results

### General characteristics of participants

Among the 39,199 participants recruited during the study period, 6685 older individuals were aged 65–79. We selected 1292 older participants ascertained with COPD using spirometry. After excluding participants with missing data (n = 259), 1033 participants were finally included. The mean age was 71.64 ± 0.14 years and 74.2% (n = 766) were men. Table 1 summarizes the participant characteristics according to the total PA levels. The weighted prevalence of high, moderate, and low PA levels was 8.3% (standard error [SE], 1.1%), 30.3% (SE, 1.7%), and 61.4% (SE, 1.9%), respectively.

**Table 1:** Participants’ characteristics according to physical activity level (n = 1033).

	High (n = 72)	Moderate (n = 289)	Low (n = 672)	*P* for trend
Age (years)	70.74 ± 0.48	71.22 ± 0.26	71.98 ± 0.19	0.008
Male sex (%)	86.6 (5.5)	81.8 (2.4)	71.7 (2.0)	0.007
BMI (kg/m^2^)	23.87 ± 0.32	23.84 ± 0.17	23.90 ± 0.12	0.956
**Smoking (%)**				0.280
Never	20.1 (5.8)	33.4 (3.1)	34.7 (2.1)	
Past	57.3 (7.1)	46.0 (3.3)	44.8 (2.2)	
Current	22.7 (5.5)	20.6 (3.0)	20.5 (1.9)	
Excessive alcohol consumption (%)	18.8 (6.0)	10.7 (2.3)	9.7 (1.4)	0.172
**Education (%)**				0.017
Middle school and lower	51.8 (7.1)	61.8 (3.4)	69.4 (2.2)	
High school and higher	48.2 (7.1)	38.2 (3.4)	30.6 (2.2)	
**Comorbidities (%)**				
Hypertension	46.7 (6.8)	59.1 (3.1)	60.0 (2.1)	0.144
Diabetes	33.7 (7.2)	22.6 (2.8)	30.8 (2.0)	0.084
Anemia	6.6 (2.9)	7.2 (1.7)	11.2 (1.5)	0.158
Asthma	3.0 (1.8)	6.6 (2.0)	6.6 (1.1)	0.535
Cardiovascular disease	9.5 (4.2)	10.9 (2.3)	13.2 (1.5)	0.606
Depression	1.2 (1.2)	3.8 (1.2)	4.8 (1.0)	0.339
Total calorie intake (kcal/day)	2233.20 ± 151.32	1953.77 ± 49.40	1778.01 ± 30.82	< 0.001
Handgrip strength (kg)	33.36 ± 0.87	31.25 ± 0.48	28.52 ± 0.37	< 0.001
FEV_1_ (L)	2.19 ± 0.06	2.17 ± 0.04	2.04 ± 0.02	0.003
FVC (L)	3.55 ± 0.10	3.42 ± 0.05	3.23 ± 0.04	0.001
Peak expiratory flow (L/s)	6.11 ± 0.19	5.84 ± 0.12	5.54 ± 0.08	0.007

Data are weighted means ± SE or weighted percentage (SE), as appropriate.

BMI, body mass index; FEV_1_, forced expiratory volume in one second; FVC, forced vital capacity.

Older COPD participants with low PA levels were significantly more likely to be aged older and female with a lower education and total calorie intake than those with high PA. Moreover, the participants with low PA were found to have lower FEV1, FVC, and peak expiratory flow as well as lower handgrip strength than those with high PA. BMI, smoking status, excessive alcohol consumption, and the presence of comorbidities did not differ across the different PA levels.

### Association between PA level and dynapenia

**Figure 1:**
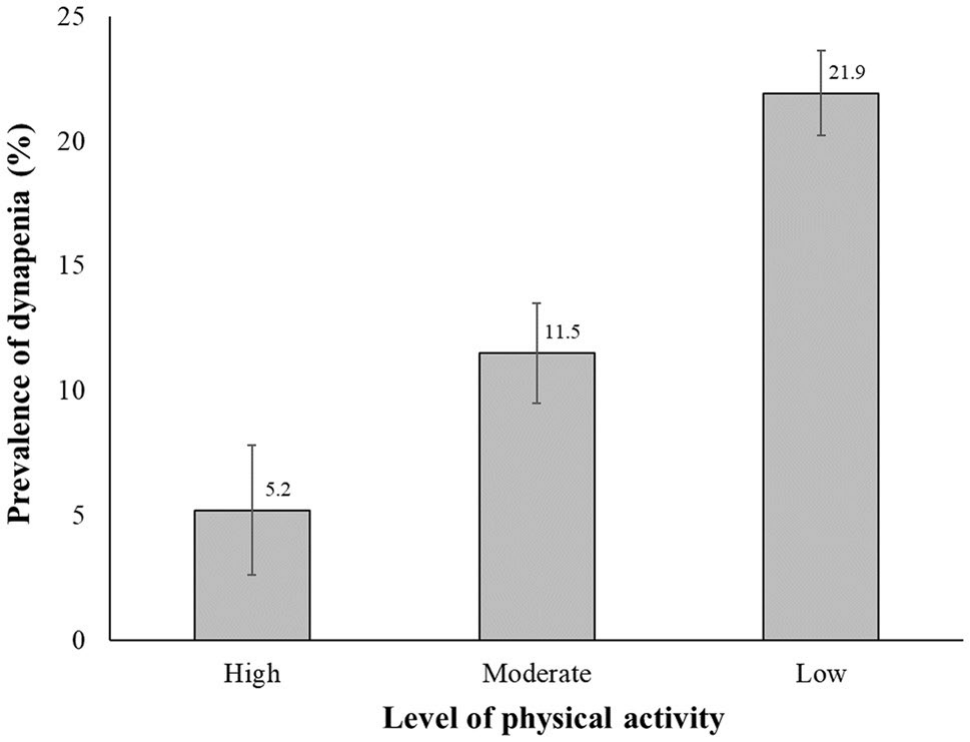
Prevalence of dynapenia according to the level of physical activity.

The weighted prevalence of dynapenia in participants with high, moderate, and low PA levels was 5.2% (SE, 2.6%), 11.5% (SE, 2.0%), and 21.9% (SE, 1.7%), respectively. (Fig. 1) Table 2 illustrates the negative association between PA level and dynapenia in older participants with COPD. The participants with moderate and high levels of PA showed a significantly lower likelihood of having dynapenia than those with low levels of PA in the minimally adjusted model (model 1 of Table 2). A negative association between PA level and dynapenia was continuously observed through further adjusted models (models 2 and 3 of Table 2). The adjusted odds ratios (ORs) for dynapenia for high and low PA levels were lower than those for moderate and low PA levels.

**Table 2:** Association of physical activity level with dynapenia.

	*Model 1*	*Model 2*	*Model 3*
Low	1.00	1.00	1.00
Moderate	0.55 (0.36–0.85)	0.54 (0.34–0.85)	0.55 (0.35–0.87)
High	0.26 (0.09–0.74)	0.29 (0.10–0.82)	0.26 (0.09–0.74)
*P* for trend	0.002	0.003	0.002

Data are odds ratios (95% confidence interval).

Model 1: adjusted for age and sex.

Model 2: based on model 1, further adjusted for body mass index, smoking, alcohol, education, hypertension, diabetes, anemia, asthma, cardiovascular disease, depression, and total calorie intake.

Model 3: based on model 2, further adjusted for Global Initiative for Chronic Obstructive Lung Disease (GOLD) stage.

### Association of physical activity level with dynapenia in groups by sex

The association between PA level and dynapenia differed between men and women. The negative association between physical activity level and dynapenia was only significant in older male COPD participants. A higher PA level in men with COPD was significantly associated with a lower odds of having dynapenia than a lower PA level in the age-adjusted model (model 1 in Table 3). The negative association between PA level and dynapenia remained significant in further adjusted models (models 2 and 3 in Table 3). In contrast, the significant associations between PA and dynapenia were not observed in older women with COPD.

**Table 3:** Association of physical activity level with dynapenia in groups by sex.

	Model 1	Model 2	Model 3
**Men (n = 766)**			
Low	1.00	1.00	1.00
Moderate	0.49 (0.29–0.85)	0.49 (0.28–0.85)	0.49 (0.27–0.88)
High	0.19 (0.04–0.80)	0.20 (0.05–0.79)	0.17 (0.04–0.65)
*P* for trend	0.003	0.003	0.002
**Women (n = 267)**			
Low	1.00	1.00	1.00
Moderate	0.70 (0.33–1.46)	0.71 (0.33–1.53)	0.74 (0.34–1.59)
High	0.48 (0.09–2.53)	0.42 (0.08–2.28)	0.42 (0.08–2.24)
*P* for trend	0.476	0.441	0.481

Data are odds ratios (95% confidence interval).

BMI, body mass index.

Model 1: adjusted for age.

Model 2: based on model 1, further adjusted for BMI, smoking, alcohol, education, hypertension, diabetes, anemia, asthma, cardiovascular disease, depression, and total calorie intake.

Model 3: based on model 2, further adjusted for Global Initiative for Chronic Obstructive Lung Disease (GOLD) stage.

### Association between PA level and dynapenia in groups by obesity status

The association between PA level and dynapenia was different by obesity status, as presented in Table 4. Specifically, in the normal-weigh group, a significant inverse association between PA level and dynapenia was observed. The ORs of dynapenia further decreased as PA level increased. Conversely, the associations between PA level and dynapenia were not significant in the obese group in the minimally adjusted model (model 1 in Table 4) and fully adjusted model (model 3 in Table 4).

**Table 4:** Association of physical activity level with dynapenia in groups by obesity status.

	Model 1	Model 2	Model 3
**Normal weight group (18.5 ≤ BMI < 25 kg/m**^**2**^**, n = 661)**			
Low	1.00	1.00	1.00
Moderate	0.44 (0.26–0.75)	0.47 (0.26–0.84)	0.48 (0.27–0.86)
High	0.20 (0.04–0.88)	0.22 (0.05–0.97)	0.21 (0.05–0.88)
*P* for trend	0.003	0.011	0.011
**Obese group (BMI ≥ 25 kg/m**^**2**^**, n = 351)**			
Low	1.00	1.00	1.00
Moderate	0.90 (0.39–2.08)	0.67 (0.31–1.48)	0.57 (0.27–1.22)
High	0.50 (0.11–2.27)	0.71 (0.14–3.62)	0.34 (0.03–3.35)
*P* for trend	0.661	0.589	0.261

Data are odds ratios (95% confidence interval).

BMI, body mass index.

Model 1: adjusted for age and sex.

Model 2: based on model 1, further adjusted for BMI, smoking, alcohol, education, hypertension, diabetes, anemia, asthma, cardiovascular disease, depression, and total calorie intake.

Model 3: based on model 2, further adjusted for Global Initiative for Chronic Obstructive Lung Disease (GOLD) stage.

## Discussion

The present study demonstrated an inverse association between PA level and dynapenia among community-dwelling older individuals with COPD. As the PA level increased, the odds for dynapenia decreased. This inverse association between PA level and dynapenia was independent of multiple sociodemographic and clinical variables; however, was influenced by sex and obesity status. An independent negative association of PA level for dynapenia was observed only in males with COPD and non-obese participants (BMI < 25 kg/m^2^). There has been considerable research investigating the relationship between PA and sarcopenia in the general older population^29,30^. However, to the best of our knowledge, the current study is the first to report a dose-dependent independent negative association between PA level and dynapenia in an older population with COPD.

Despite the lack of studies for the COPD population, in the general older population, multiple studies from different ethnic groups and meta-analysis have indicated an inverse association between PA and sarcopenia^29,30^. Our results documenting a negative association between PA level and dynapenia among older individuals with COPD is consistent with those of previous studies in the general older population. In contrast, a substantial amount of research has documented the association of PA levels with mortality and exacerbations in individuals with COPD^31^. Nevertheless, in patients with COPD, evidence is still lacking on the factors associated with the level of PA. Dynapenia can be associated with PA level owing to possibly better prediction of disability and physical performance than the muscle mass^6,7^. Experimental studies support the protective role of PA on age-related changes in the sarcopenic muscle by modulating muscle metabolism, inflammation, and musculotendinous architecture at the intracellular level^32^.

The result that the likelihood of dynapenia decreases as the PA level increases suggests a potentially beneficial role of PA on age-related muscle weakness in the COPD population. This dose-dependent relationship between PA level and dynapenia observed in the present study is in agreement with the results of previous studies performed in community-dwelling older people^33,34^. Foong et al. showed a cross-sectional dose–response relationship between the amount and intensity of PA and lower extremity muscle strength in 636 non-hospitalized older people^34^. Considering the clinical significance of PA and dynapenia in older patients with COPD, clinicians should make efforts to increase PA levels and reduce dynapenia. From a clinical perspective, for the community-dwelling older population, interventions that increase PA levels through aerobic activity can be easier than those on the muscle strength to reduce dynapenia or sarcopenia.

The current study demonstrated a significant difference in the mean hand grip strength according to PA level (Table 1) as well as an independent association between dynapenia and PA level. Although dynapenia has not been considered, a few previous studies have analyzed the handgrip strength as a continuous variable to assess the association between handgrip strength and PA in patients with COPD^14,35^. However, the findings of the earlier studies that PA was not associated with the handgrip strength in patients with COPD disagree with those of our study^14,35^. Contrarily, a linear and positive association between PA and handgrip strength reported in 66,582 older adults in the United Kingdom is similar to the results of our study^36^. The discrepancies between our study and previous studies performed in COPD population may be owing to the different methods of evaluating PA level across studies or the effects of potential confounders. Furthermore, the hospital-based study design and small sample size of previous studies in participants with COPD may hinder obtaining sufficient statistical significance.

The associations between PA and dynapenia were different in men and women. Whereas higher PA levels were independently associated with lower odds of dynapenia in men, this significant association was not observed in women. Hormones may contribute to these sex differences in the associations between PA and dynapenia. It has previously been suggested that dynapenia process with aging differs between men and women and the reduction of sexual hormones may play an import role in maintaining skeletal muscle mass^37^. Sex hormonal status has a significant impact on muscle mass in elderly men, but not in elderly women^38^. Decreased testosterone and insulin-like growth factor-1 levels with aging results in a gradual decline in muscle mass in men^39^. Although women also experience muscle loss and changes in muscle strength with aging, it is known that women could maintain relatively good muscle function even later in their lifetime when compared to men. Furthermore, there is some evidence that lack of PA during a men’s entire life is a contributing factor to the loss of muscular mass. In contrast to men, muscle volume and performance in elderly women is independent of physical activity^18^.

Obesity affects muscle strength in healthy adults^20^ and the degree of obesity explains 2%–16% of the variability of muscle strength^40^. Furthermore, PA in older adults was also negatively affected by obesity^19^. Unlike the general population, obese individuals with COPD may present better prognosis with lesser lung hyperinflation than COPD patients with normal body composition owing to the obesity paradox^41,42^. A recent cross-sectional study suggested that abdominal obesity may have beneficial effects on physical functioning^43^. Nevertheless, other studies found that obesity has a negative impact on PA in daily life and exercise capacity in patients with COPD^15,16^. Obese patients with COPD showed significant exercise-induced limb fatigue, despite the greater limb strength compared with healthy controls^44^. Therefore, we hypothesized that obesity may influence on the association between PA level and dynapenia in older patients with COPD. Normal-weight individuals with moderate and high levels of PA had a significant reduction in the odds of dynapenia in multivariable-adjusted models. However, older COPD patients with obesity did not benefit from the reduction of dynapenia risk.

Evidence-based clinical practice guidelines (CPGs) have been developed to perform best practice for patients with COPD in different countries. However, there is a lack of specific recommendations, including the type, context, intensity, duration, and frequency of PA among those CPGs^45^. Furthermore, the optimal dose of PA sufficient to reduce the risk of dynapenia in the older population with COPD remains to be determined. The results of our study suggest that aerobic PA above moderate levels suggested by the WHO would be beneficial to reduce the likelihood of dynapenia. However, these favorable effects of PA on dynapenia may not be applicable to older COPD patients with obesity as well as older women with COPD. Prospective studies regarding the longitudinal effects of PA level on dynapenia are necessary in the future.

The large nationally representative cohort data of the community-dwelling older population with COPD is the strength of the present study. Furthermore, we adjusted multiple established and potential confounders for PA and muscle strength, including obesity, lung function, and nutrient intake, to suggest an independent association. Additionally, we performed stratification analyses by sex and obesity status for evaluating the influencing factors on the prevalence of dynapenia among patients with COPD by PA level. However, several limitations of this study should be recognized. The cross-sectional design makes it difficult to establish a causal relationship between the variables. Further longitudinal studies or randomized controlled trials are needed to validate causality. Second, the PA level was evaluated using a self-reported questionnaire, which has disadvantages such as memory recall bias. However, GPAQ has been reliably used to evaluate detailed PA in earlier studies^26^. Third since prebronchodilator FEV1/FVC ratio criteria were used to diagnose COPD, the possibility of overestimating the number of participants with COPD by inclusion of asthmatic participants cannot be excluded.

In conclusion, low PA levels are independently associated with increased likelihood of dynapenia in older patients with COPD. There is an additional benefit of decreasing odds of dynapenia as the PA level increases. Regular assessment of PA levels in older adults with COPD may be helpful to monitor muscle function or vice versa. Sex and obesity status greatly influence the association between PA and dynapenia. The factors associated with dynapenia or PA in female COPD patients and COPD patients with obesity should be investigated in the future studies.
